# Big data analysis techniques to address polypharmacy in patients – a scoping review

**DOI:** 10.1186/s12875-020-01247-1

**Published:** 2020-09-03

**Authors:** D. Wilfling, A. Hinz, J. Steinhäuser

**Affiliations:** Institute of Family Medicine, University Hospital Schleswig-Holstein, Campus Lübeck, Ratzeburger Allee 160, 23538 Lübeck, Germany

**Keywords:** Big data, eHealth, Polypharmacy

## Abstract

**Background:**

Polypharmacy is a key challenge in healthcare especially in older and multimorbid patients. The use of multiple medications increases the potential for drug interactions and for prescription of potentially inappropriate medications. eHealth solutions are increasingly recommended in healthcare, with big data analysis techniques as a major component. In the following we use the term analysis of big data as referring to the computational analysis of large data sets to find patterns, trends, and associations in large data sets collected from a wide range of sources in contrast to using classical statistics programs. It is hypothesized that big data analysis is able to reveal patterns in patient data that would not be identifiable using conventional methods of data analysis. The aim of this review was to evaluate whether there are existing big data analysis techniques that can help to identify patients consuming multiple drugs and to assist in the reduction of polypharmacy in patients.

**Methods:**

A computerized search was conducted in February 2019 and updated in May 2020, using the PubMed, Web of Science and Cochrane Library databases. The search strategy was defined by the principles of a systematic search, using the PICO scheme. All studies evaluating big data analytics about patients consuming multiple drugs were considered. Two researchers assessed all search results independently to identify eligible studies. The data was then extracted into standardized tables.

**Results:**

A total of 327 studies were identified through the database search. After title and abstract screening, 302 items were removed. Only three studies were identified as addressing big data analysis techniques in patients with polypharmacy. One study extracted antipsychotic polypharmacy data, the second introduced a decision support system to evaluate side-effects in patients with polypharmacy and the third evaluated a decision support system to identify polypharmacy-related problems in individuals.

**Conclusions:**

There are few studies to date which have used big data analysis techniques for identification and management of polypharmacy. There may be a need to further explore interdisciplinary collaboration between computer scientists and healthcare professionals, to develop and evaluate big data analysis techniques that can be implemented to manage polypharmacy.

## Background

Polypharmacy is an essential challenge especially in older and multimorbid patients. The term polypharmacy was used over one and a half centuries ago to refer to issues related to the consumption of multiple drugs and excessive drug use [1]. Intake of five or more medications is a commonly used definition of polypharmacy. It has been shown that an intake of at least five medications significantly increases the risk of adverse events, such as falls, frailty, disability and mortality [2, 3]. Furthermore, drug-drug interactions are common with the use of multiple drugs and the prescription rate of potentially inappropriate medications raises. Neuropsychological problems like delirium, acute renal failure and hypotension are the most common unwanted side effects [2].. Furthermore, polypharmacy can lead to problems with medication adherence, especially in older adults if associated with visual or cognitive decline as well as aging, resulting in unwanted outcomes such as treatment failure or hospitalizations [4]. The prevalence of polypharmacy at hospital admission in various countries was reported to be between 20 and 60% [5–7] and it was recently reported that rehospitalization results in a significant increase in the number of drugs given to patients at discharge. Because of the reported risk of adverse drug reactions in patients receiving polypharmacy, optimal drug prescription is important for these individuals [8, 9]. EHealth, including the use of electronic devices and systems, should be integrated into healthcare because of their potential to improve the treatment of patients, especially multimorbid patients with polypharmacy [10, 11]. Big data analysis techniques as a part of eHealth were introduced in 1997 [12], and were defined by the “3Vs”: increasing volume of data, high velocity of data, and variety of data [13–15]. In the following we use the term analysis of big data as referring to the computational analysis of extremely large data sets to find patterns, trends, and associations in data collected from a wide range of sources in contrast to using classical statistics programs [12].

According to current recommendations the main advantage of big data analysis is the ability to identify new contexts and patterns in patient data that would go undetected using conventional methods of data analysis [16].

Data on drug prescription is frequently embedded in free-text fields in electronic health records [17, 18]. To extract free-text information, manual coding is necessary, which means that a human must read the free-text and assign codes to it manually using a defined set of coding rules [18]. This procedure is very time and labour intensive. Electronic health records texts have been analysed automatically using techniques such as natural language processing for a variety of purposes, e.g. the identification of drugs [19, 20]. However, attempts to develop and validate techniques for characterising meta-data such as polypharmacy have not been made [21].

It is not yet known if analysis of big data is potentially useful in the identification of patients with polypharmacy and thus the reduction of the risk of adverse events caused by multiple medications. The aim of this review was to evaluate whether big data analysis techniques already exist that can help identify patients consuming multiple drugs and to assist in the reduction of polypharmacy in patients.

## Methods

This review aimed to identify, appraise and summarize the current evidence on the use of big data analysis in identifying polypharmacy in patients. Established methodological frameworks for systematic evidence syntheses [22] and the preferred reporting items for Scoping Reviews [23] were used. No study protocol was registered.

### Search methodology

The search strategy was defined by the principles of a systematic search, using the PICO scheme, and implied free-text keywords and Medical subject headings (Mesh terms) by two reviewers. A computerized search was conducted in February 2019 in the PubMed, Web of Science and Cochrane Library databases. A complex search strategy was developed in order to detect all areas of big data analysis. Major search terms for all databases are represented in Table 1. Relevant grey literature was located using a systematic search on Google Scholar. For this search the terms “big data” AND “polypharmacy” were used. Furthermore, we scrutinized reference lists of studies included and relevant reviews identified through the search. The results of the searches were imported into the web service Covidence (www.covidence.org) which was used for the entire review process. We updated the search in May 2020.

**Table 1:** Search terms

big data *OR*	*AND*
health analytics *OR*	
healthcare informatics *OR*	
electronic health records *OR*	
databases *OR*	
data collection system *OR*	
electronic data capture *OR*	
data management system *OR*	
deep learning *OR*	
electronic medical record *OR*	
machine learning *OR*	
medical data *OR*	
huge data *OR*	
electronic patient record *OR*	
datamining *OR*	
data analysis *OR*	
reinforcement learning *OR*	
decision support system *OR*	
predictive analytics *OR*	
reasoning *OR*	
inference *OR*	
polypharmacy [MeSh] *OR*	*AND*
drug therapy *OR*	
inappropriate prescribing *OR*	
inappropriate medication *OR*	
over-prescribing *OR*	
suboptimal prescribing *OR*	
multiple medication* *OR*	
multiple medicine* *OR*	
multiple drug* *OR*	
many medication* *OR*	
many medicine* *OR*	
many drug* *OR*	

### Study selection

All scientific articles evaluating big data analysis techniques to identify patients consuming more than five drugs were included, irrespective of study design and publication year. For inclusion, studies had to be in English or German. The University of Lübeck operates the “Center for Open Innovation in Connected Health (COPICOH)”, of which the authors of this review are members, working alongside computer scientists and researchers from other health care disciplines. Consensus meetings were held with other members of COPICOH in order to discuss articles if the authors were uncertain if an article should be included or excluded.

It was decided that studies that used standard statistical methods, for example large cohort studies that examined data from electronic health records, were not deemed eligible. Studies focusing on identifying new drug-drug interactions or new drug combinations were also excluded. Studies addressing big data analysis techniques to identify drug interactions or adverse drug events in patients on multiple medications were included. Although we originally determined that patients must be taking five medications or more to meet the criteria, we decided to include studies with patients taking three medications or more due to a lack of studies. Inclusion and exclusion criteria are shown in Table 2.

**Table 2:** Inclusion and exclusion criteria

*Inclusion*	*Exclusion*
English-language articles German-language articles	Articles in other languages
All kinds of studies evaluating big data analysis techniques	Big cohort studies examining data from electronic health records
Big data as computational analysis of extremely large data sets to find patterns, trends and associations	Studies using classical statistics programs
Patients with polypharmacy (more than 3 medications)	Patients consuming less than 3 medications
Big data analysis techniques to identify patients with polypharmacy as well as drug interactions or adverse drug events in patients on multiple medications were included	Studies focusing on identifying new drug-drug interactions or new drug combinations

Two independent reviewers (DW, AH) assessed titles and abstracts from all search results to identify eligible studies. After selection of potentially relevant articles, full reports were obtained and assessed for inclusion and exclusion criteria. Any disagreement on the eligibility of studies was resolved through discussion to reach consensus or, if required, by involving a third experienced review author (JS). We used the details from the selection process in Covidence to complete the PRISMA flow diagram.

### Data extraction

Data from each included study was extracted by one reviewer, with the accuracy of extraction being independently cross-checked by a second reviewer. In case of disagreements or discrepancies, a third review author was called upon to reach consensus. The validity of studies was evaluated based on the judgement of two independent researchers (DW, AH). The data was extracted into standardized tables, including publication year, country of origin, aim of the study, number of examined datasets, method of data analysis used and outcomes.

## Results

### Description of studies included

**Fig. 1:**
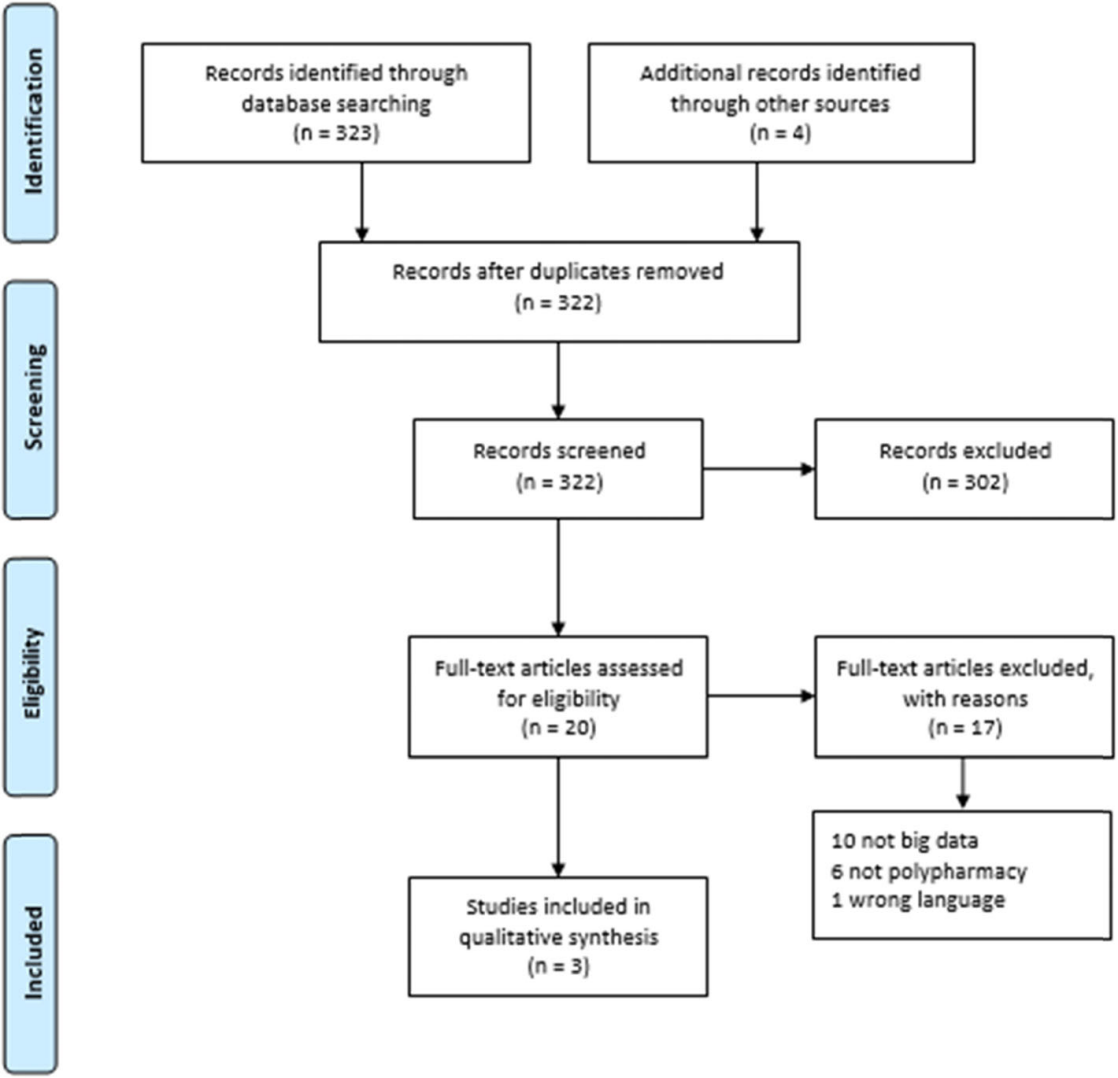
PRISMA flow chart

A total of 327 studies were identified through database searches. After removing duplicates, the final dataset consisted of 322 articles. Following title and abstract screening, 302 records were removed. 20 full texts were screened and finally three studies were included in the review. Main reasons for exclusion were not meeting the criterium of big data analysis (*n* = 10), polypharmacy (*n* = 6) and not being in English or German (n = 1). All included studies were published in English. Two studies were conducted in the US and one in the UK. For better traceability, the entire screening process is visualized using the PRISMA flow chart (Fig. 1). The results of the data extraction are summarized in Table 3.

**Table 3:** Characteristics of included studies

Author, Year	Country	Aim	No. of used observations	Method of data analysis	Outcome
Keine et al. 2019 [24]	USA	Evaluating a precision medicine platform to identify a multitude of polypharmacy problems in people with dementia and mild Alzheimer’s disease through the creation of personalized, multidomain treatment plans	295 patients with a family history of Alzheimer’s disease or mild cognitive decline	Clinical decision support software (CDSS) with machine-learning algorithms	The system was able to identify a multitude of polypharmacy problems that individuals are currently facing.
Kadra et al. 2015 [25]	UK	Extracting antipsychotic polypharmacy data from structured and free-text fields in electronic health records	7201 patients with serious mental illness	Combination of natural language processing and a bespoke algorithm.	Individual instances of antipsychotic prescribing, 2 or more antipsychotics prescribed in any 6 week window; antipsychotic co-prescribing for 6 months
Duke et al. 2010 [26]	USA	Creating a decision support system tailored to the evaluation of adverse reactions in patients on multiple medications	16,340 unique drug and side-effect pairs, representing 250 common medications	A numeric score was assigned to reflect the strength of association between drug and effect. Based on this score, the system generates graphical adverse reaction maps for any user-selected combination of drugs.	This tool demonstrated a 60% reduction in time to complete a query (61 s vs. 155 s, *p* < 0.0001) with no decrease in accuracy

### Analysis of big data for patients with polypharmacy

Kadra et al. 2015 [25] extracted antipsychotic data (APP) from two big healthcare providers in Europe, containing structured as well as free-text labels. This data was supplemented by pharmacy records to estimate both the prevalence of APP and prescription patterns.

All patients with a diagnosis of schizophrenia, schizoaffective disorders or bipolar disorders who received care between January and June 2012 were included and prescription schemes for a period of six months were considered. Because antipsychotic medication data contained free-text labels, an NLP (natural language processing) information extraction application software was developed using General Architecture for Text Engineering (GATE) [25, 27]. Case records were screened for whether two or more antipsychotic drugs were prescribed within a period of six weeks between January and June 2012, defined as baseline polypharmacy (t0). Kadra et al. 2015 [25] defined long-term APP as the use of two or more antipsychotics for six or more months and therefore all patients with baseline polypharmacy were screened again six months later (t1). To guarantee generalizability, the APP algorithm was verified following an iterative validation process. Because the NLP application combined with the APP algorithm showed significant results, it can be assumed that patients were correctly identified as being prescribed APP. This approach is an effective combination of natural language processing and a bespoke algorithm for extracting APP data. It was possible to identify polypharmacy from electronic mental health records using this approach. Furthermore, the extracted data can be used to characterize patterns of polypharmacy over time, including different drug combinations, trends in polypharmacy prescription, predictors of polypharmacy prescription and the impact of polypharmacy on patient outcomes (e.g. mortality or physical health consequences) [25].

A decision support system to evaluate side effects in patients with polypharmacy was introduced by Duke et al. 2010 [26]. After selecting 250 commonly used medications, Structured Product Labels (SPL’s) were developed. By using and combining manual and natural language processing techniques, it was possible to extract the side effects for each SPL [28]. In a second step, a scoring system to identify the relationship between each drug and associated adverse events was developed. For this purpose, single numeric values were used and 39 different algorithms were needed. In total, 16,340 medications and adverse events were extracted and the most common side effects of each drug was presented by bar graphs (with the bar length proportional to the calculated score for each side effect). It was also possible to filter the results for specific side effects that were of interest [26, 28]. The developed scoring system was evaluated in a pilot study involving 24 physicians. For evaluating speed and accuracy of the developed tool, two sample clinical tasks including a patient description, a medication list and four hypothetical side effects were presented. Physicians were then asked one of the following two questions: which of the patient’s drugs are known to cause this reaction; or which one drug is most likely to cause this reaction. Time and answer were recorded and finally the results were compared with the results from tool developed. Results showed that using the application is 60% faster in searching adverse events compared to traditional drug information resources. Furthermore, physicians were very satisfied with the developed tool and rated its usability as very high [28].

Another decision support system was evaluated by Keine et al. 2019 [24]. The aim of this system was to identify problems of polypharmacy in individuals, such as drug-drug interactions (DDIs), drug-genome interactions (DGIs), and drug-diet interactions. The clinical decision support system using machine-learning algorithms created recommendations to help physicians with medication management in order to improve clinical decision making and patient safety. The algorithms are capable of parsing interactions, rating them based on input from opensource databases, and recording all interactions in the treatment plan. This enables physicians to review a patient’s medication plan in an easy way. The decision support system was evaluated using 295 individuals aged 65 and older. Of the 295 individuals, 97.59% were on at least one medication, with an overall mean of 11.5 medications per person, with 83.66% of them on five or more medications. Additionally, many interactions were identified [24].

## Discussion

The aim of this review was to evaluate whether there are existing big data analysis techniques that can help identify patients consuming multiple drugs and assist in reducing polypharmacy in patients. We identified only three articles using this approach to identify cases of polypharmacy and to avoid the side-effects of multiple medications. Although polypharmacy is defined as taking five or more medications, this review includes studies with patients taking at least three medications. The number of studies addressing the use of big data analysis in patients with polypharmacy is very small so we have no indication that the redefinition of the inclusion criteria was likely to have a negative impact on the results or would have changed our conclusions.

Kadra et al. 2015 [25] presented an effective combination of natural language processing and a bespoke algorithm for extracting APP data. They were able to not only identify polypharmacy using electronic mental health records but also different drug combinations, trends in polypharmacy prescription, predictors of polypharmacy prescription and the impact of polypharmacy on patient outcomes [25]. This approach has to be examined over a longer period of time and further validated, including a broader variety of medications.

Two studies [24, 26] were identified as focusing on uncovering drug-drug interactions in patients on multiple medications. The developed scoring system from Duke et al. 2010 [26] was evaluated in a pilot study and the results showed that using the application is 60% faster in searching for adverse events compared to traditional drug information resources. Furthermore, physicians were very satisfied with the tool developed and rated its usability as very high [29]. This approach has also to be validated further and evaluated in the clinical setting.

The third study [24] included in this review also addresses interactions in medication plans. The results of the evaluation showed that older populations have a high medication burden and therefore medication management for polypharmacy is a challenging task. The system developed was able to identify a multitude of polypharmacy problems that individuals are currently facing [24].

Although the results are promising overall, the identified approaches have to be examined and validated further. In addition, a clear definition of big data is missing from all studies. Similar results have been shown in another recently published review aiming to evaluate big data analysis for multimorbid patients [30]. Based on the results of our review and the results from other studies, there is a lack of big data analysis integration in health care [30–33]. Through our screening process, we identified two other studies [34, 35] aiming to develop big data analysis to identify new drug-drug interactions or effective drug combinations in cancer therapy, but it did not address patients with polypharmacy. Thus, a lack of big data analysis implementation in health care for addressing polypharmacy was also identified.

None of the other identified reviews on big data analysis included a definition of big data [24, 29, 30]. There is an assumption that big data analysis represents a big opportunity for healthcare. The use of big data analysis techniques is growing quickly, both in clinical medicine as well as health care administration. The analysis of health care data has the potential to reduce treatment costs, avoid preventable diseases and improve the quality of care [12]. In terms of polypharmacy, this would mean a reduction of multiple medications in patients, prevention of drug-drug interactions and improving quality of life.

This review has clearly shown that there is a need to further develop and evaluate big data analysis techniques and algorithms in health care and to implement them in the clinical setting.

### Limitations

#### Strengths and limitations

To the best to our knowledge, this is the first review addressing analysing big data in the treatment of patients with polypharmacy. To minimize bias, the whole screening and data extraction process was conducted by two independent researchers. There may be a risk of publication bias because researchers working in the field of big data analysis may be publishing their results in journals other than those used by health professionals. We conducted an additional search on Google Scholar in order to identify articles that might not be indexed in PubMed. A limitation of this review is that only a very small number of studies (*n* = 3) are included. A further limitation is that we did not contact study authors and experts in the field and did not ask about unpublished or ongoing studies.

## Conclusion

We identified only one study that used a big data approach to identify patients with polypharmacy and two studies that used big data approaches to avoid side effects in patients with multiple medications. A clear definition for big data analysis was missing from all studies. Big data analysis techniques and algorithms currently exist in other contexts, but they are rarely used in healthcare. One possible way to improve implementation of big data analysis may be to further develop interdisciplinary research environments involving computer sciences and health care professionals. This could allow for development and evaluation of big data analysis techniques in clinical settings, for example in the management of polypharmacy.

## Supplementary information

**Additional file 1.** Full electronic search strategy in PubMed.

## Data Availability

The complete search strategy is available on request.

## Abbreviations

NLP: Natural language processing
GATE: General Architecture for Text Engineering
APP: Antipsychotic data
SPL: Structured Product Label
DDI: Drug-drug interaction
DGI: Drug-genome interaction
