# Supplementary material for: Big data analysis techniques to address polypharmacy in patients – a scoping review
Source: BMC Fam Pract. 2020 Sep 3;21:180. doi: 10.1186/s12875-020-01247-1 (PMC7472702; doi:10.1186/s12875-020-01247-1)
Supplement: Supplementary file 1 — Additional file 1. Full electronic search strategy in PubMed. [file 12875_2020_1247_MOESM1_ESM.docx]

**(((((((((((((((((("Big data"[Title/Abstract]) OR "Health analytics"[Title/Abstract]) OR "Healthcare informatics"[Title/Abstract]) OR "Electronic health records"[Title/Abstract]) OR "data collection system"[Title/Abstract]) OR "electronic data capture"[Title/Abstract]) OR "data management system"[Title/Abstract]) OR "deep learning"[Title/Abstract]) OR "electronic medical record"[Title/Abstract]) OR "machine learning"[Title/Abstract]) OR "medical data"[Title/Abstract]) OR "electronic patient record"[Title/Abstract]) OR "datamining"[Title/Abstract]) OR "reinforcement learning"[Title/Abstract]) OR "decision support system"[Title/Abstract]) OR "predictive analytics"[Title/Abstract]) OR "Inference"[Title/Abstract])) AND ((((((((((((polypharmacy[MeSH Terms]) OR "drug therapy"[Title/Abstract]) OR "inappropriate prescribing"[Title/Abstract]) OR "Inappropriate medication"[Title/Abstract]) OR "over-prescribing"[Title/Abstract]) OR "suboptimal prescribing"[Title/Abstract]) OR "multiple medication*"[Title/Abstract]) OR "multiple medicine*"[Title/Abstract]) OR "multiple drug*"[Title/Abstract]) OR "many medication*"[Title/Abstract]) OR "many medicine*"[Title/Abstract]) OR "many drug*"[Title/Abstract])**

Supplement 1: Full electronic search strategy in PubMed
